# Implementation research for today's HIV response: from theory to applied insights

**DOI:** 10.1002/jia2.26305

**Published:** 2024-07-05

**Authors:** Bohdan Nosyk, Eleanor Magongo Namusoke, Anne Trolard, Elvin H. Geng

**Affiliations:** ^1^ Faculty of Health Sciences Simon Fraser University Burnaby British Columbia Canada; ^2^ Ministry of Health, Government of Uganda Kampala Uganda; ^3^ Center for Dissemination and Implementation Institute for Public Health Division of Infectious Diseases Department of Medicine School of Medicine Washington University in St. Louis St. Louis Missouri USA

Global progress over the past 20 years has turned the tide on the HIV epidemic. Many countries are close to, and some have even reached, the UNAIDS 90‐90‐90 (and now 95‐95‐95) goals. Looking into the future, however, progress now requires not only continued attention, but a shift in scientific and strategic directions. Programmes must advance *equitable reach* to ensure that HIV prevention and treatment services meet the needs of populations and contexts that are outside of mainstream health services. We must shift from the continued rapid growth of capacity to *sustainable systems* embedded within policy and economic commitments around the world. Finally, the HIV response must evolve from a sole focus on HIV towards integrated services for comorbid conditions, both in persons living with HIV as well as to contribute to a global push for universal health coverage for all persons.

Implementation research is well‐positioned to address this new generation of challenges and is, therefore, needed more than ever in the scientific response to HIV today. The growing prominence of implementation research for HIV is reflected in the assembly of this collection of articles for this supplement in the Journal of International AIDS Society (JIAS) on *Implementation research and the HIV response: Taking stock and charting the way forward*, as well as the growing number of funding opportunities, publication venues and professional settings which focus on implementation research. Implementation research has been defined as methods to promote the systematic uptake of research findings and other evidence‐based practices into routine practice to improve the quality, reach and sustainability of health services [1]. The scientific questions we seek to answer today are fundamentally questions about implementation: how to achieve greater and more equitable reach; how to sustain services in a changing economic and policy environment; how to integrate HIV services into wider public health structures. At the same time, the research needs of the HIV community also provide a critical testing ground to assess and refine implementation science methods to optimally deliver actionable insights for real‐world problems and help us achieve greater epidemic control. Is the HIV research community up to the task?

This supplement responds strongly in the affirmative. This solicitation received over 100 submissions, with studies conducted in Africa, Asia, Europe, and North and South America. The 12 ultimately included cover a wide range of interventions—partner services, HIV self‐testing, long‐acting injectable antiretroviral therapy (ART) and stepped care for ART retention, and pre‐exposure prophylaxis (PrEP)—as well as responses at the population level and for key populations, such as infants and young women. The articles also make use of a range of frameworks, from the Consolidated Framework for Implementation Research (CFIR) to Normalization Process Theory (NPT), as well as ways of classifying strategies (using the Expert Recommendations for Implementing Change) and adaptations (using the Framework for Reporting Adaptations and Modifications‐Enhanced [FRAME]). Together, they offer concrete examples of how implementation science methods can be used to produce actionable research findings and, in some examples, meaningfully shift clinical practice at the population level.

Several of the studies focus on innovative strategies to extend the reach and efficiency of HIV testing—a critical step needed to close remaining gaps in the public health response. Even though the number of people living with an HIV diagnosis globally has risen rapidly over the last decade, as much as 30% in some settings still present with advanced disease at diagnosis, motivating renewed efforts to extend the reach of HIV testing. The observation that people who most need particular health services are often the last to receive them was coined the “inverse law” in 1971 [2]. Several articles explore how to overcome this tendency, using both technological progress (e.g. self‐testing kits) and novel ways to distribute testing outside of traditional brick‐and‐mortar health services.

Other studies use innovative methods to extend reach through leveraging social networks. Through a large cluster‐randomized trial, Roy Paladhi et al. [3] demonstrated that distributing self‐testing kits to partners of people newly diagnosed with HIV was equivalent to the standard of care in which in‐person HIV testing is offered to contacts, and thus offering a route to greater efficiency in partner‐assisted services. Such innovative approaches leveraging interpersonal networks (e.g. social networks) to deliver new testing technologies should also provoke considerations for screening for other chronic conditions (e.g. diabetes, hypertension). Sharma et al. [4] examined extending partner services to partners of partners, and showed programmatic approaches that leverage sexual networks to detect new cases are feasible and useful.

The potential for internet‐based strategies in Asia for reach was highlighted by the paper from Nguyen et al. [5] who reported on the successful implementation of a web‐based HIV self‐testing programme through a population‐based observational study of over 17,000 individuals in Vietnam. The study provides a detailed examination at a subnational level of how to carry out a vast expansion of HIV testing, in this case, mediated by the internet, so that a testing programme need not contact testers in person, thus potentially reaching a segment of people who are reluctant to engage with standard health services.

Three papers in this supplement provide key information about the implementation and integration of novel HIV interventions into practice environments. Each is guided by implementation research frameworks that help connect findings with wider literature (including those outside of HIV). Vanhamel et al. [6] used extended Normalization Process Theory (NPT) to explain PrEP integration into HIV clinics in Belgium through interviews with clinic staff and observations. This study shows that wider adoption of a novel intervention is not simply replication at scale, but instead an adaptive process where individual clinics must be given sufficient leeway to innovate. Specifically, they found that both relational and normative restructuring (i.e. changes in the rules and relationships) are shaped by the existing potential and capacity at the facility. For example, they found that lower‐volume clinics integrated PrEP services into existing workflows, whereas high‐volume clinics created new procedures for PrEP services (such as grouping all PrEP patients on 1 day of the week). In a study focused on integrating long‐acting injectable antiretroviral formulations into HIV care in the United States, Nguyen et al. [7] used CFIR to conduct a cross‐sectional survey of 38 clinics. The study found clinics were most interested in technical assistance to address workflow development, payor challenges, staffing shortages for patient coordination and demand generation. The findings underscore the need for implementation, but through a process to find an approach that works within each setting. Chapuma et al. [8] used a narrative synthesis to identify failure points in early infant diagnosis and treatment (using deductive coding from CFIR), as well as Proctor's actor, action, action target framework [9] to develop concrete recommendations directed at policymakers, providers and patients.

Another important role of implementation research in the HIV response is to provide methods for developing implementation strategies so that actions taken to implement meet the desires of providers and patients, and, therefore, are more likely to be taken up. The notion of “preferences” is drawn from economic theory suggesting that humans make rational trade‐offs under constraints (time, money) to decide on the goods and services they consume, and methods to illuminate these preferences are increasingly used in HIV research [10]. Importantly, the idea of “preferences” is qualitatively different from acceptability because it places an individual's relationship to any good or service not as a relationship to that particular product, but rather in the context of their other potential choices (and costs). Mugambi et al. [11] use one such method, a discrete choice experiment, to assess the attributes that pregnant women want from HIV prevention services delivered in pharmacies. Interestingly, women found the range of services available (e.g. PrEP, testing and partner care) to be desirable, and were willing to give up other conveniences (such as location and cost) for more diversity in services. Pregnant women in Kenya chose *choice*, and that choice itself may create demand.

Velloza et al. [12] drew from human‐centred design [13]—another method that should be applied more frequently in implementation research in design of services—to adapt a peer counselling approach to PrEP delivery settings in South Africa. Design methods begin not with a cognitive technique, but an affective one—*empathy*. Empathic engagement between designers and end users enables truly collaborative co‐creation of solutions. In this paper, the design was used to adapt a mental health service originally from Zimbabwe. The article by Tan et al. [14] takes a complementary approach. Instead of end‐users as research partners, however, this paper positions “citizens” working in advocacy capacities as drivers of scientific standards. Chhun et al. [15] provide a report that applies such new standards—FRAME in this case [16, 17]—to provider perceptions of improved implementation of a stepped care intervention for ART retention in youth, carefully tracking adaptations to clinical service approach in Kenya.

Simulation modelling for decision analysis and cost‐effectiveness offers great promise for implementation and to date has been underutilized in the field of implementation research addressing HIV [18]. Enns et al. [19] demonstrated the utility of simulation modelling to project potential outcomes of a selection of evidence‐based implementation interventions in HIV testing and PrEP. This study highlights the scalability of interventions as a key determinant of their impact at the city level, though modelling can otherwise be used in the pre‐implementation stage to highlight and contrast key aspects of the potential reach, adaptation and maintenance of an intervention necessary to produce desired outcomes, especially over the longer term. This methodology is primed for further growth as a valuable addition to the implementation science toolbox.

Finally, to round out the issue, we have included a piece that takes stock of the global implementation research landscape. Lujintanon et al. [20] call attention to gaps in the applied implementation research literature, highlighting that strategies most often target patients or providers, and that approaches that act at a higher level on processes and systems—and mostly at the level of policymakers—often are lacking. This raises a question that has been posed in this Journal before as a challenge to the field of implementation science, where addressing structures and systems as well as policies must not be neglected simply because our most rigorous methods (e.g. trials) are more difficult to use at higher levels of the health system [21].

The public health response to HIV has adapted to decades of change and proven to be resilient, but once again must innovate to meet today's challenges. After the global financial crisis in 2007–2008, the global commitment to the HIV response defied expectations. While public health overall deteriorated during the global COVID‐19 pandemic, HIV treatment programmes around the world managed to continue delivering lifesaving therapy. New challenges such as climate change, economic insecurity, and increasing criminalization in some regions, are coming to destabilize the HIV response. Our work is far from done. Embracing implementation research represents one potential way to meet the challenges of the day to advance a more nimble, person‐centred and efficient response. The studies in this supplement demonstrate successful steps toward realization of the use of scientific tools from implementation science for solving real‐world contemporary challenges in HIV treatment and prevention. The biomedical interventions developed in the field of HIV represent some of the most game‐changing innovations in medicine this century [23, 24]. Can we use implementation science to create similar game‐changing innovations in the way we prevent, diagnose, engage, and treat people living with and affected by HIV? We hope this supplement provides an answer by featuring the exciting, still‐developing field of implementation science, and highlights its application to our continued efforts in tackling the global HIV epidemic.

## COMPETING INTERESTS

The authors declare no competing interests.

## AUTHORS’ CONTRIBUTIONS

EHG conceptualized and co‐wrote the first draft; BN conceptualized and co‐wrote the first draft; EMN made substantial contributions to the revision; AT make substantial contributions to the revised and final drafts.

## Data Availability

Data sharing is not applicable to this article as no datasets were generated or analysed during the current study.
